# Stability lies in flowers: Plant diversification mediating shifts in arthropod food webs

**DOI:** 10.1371/journal.pone.0193045

**Published:** 2018-02-16

**Authors:** Marcelo Mendes Haro, Luís Cláudio Paterno Silveira, Andrew Wilby

**Affiliations:** 1 Laboratório de Entomologia, Estação Experimental de Itajaí, Empresa de Pesquisa Agropecuária e Extensão Rural de Santa Catarina (Epagri), Itajaí, Santa Catarina, Brazil; 2 Departamento de Entomologia, Universidade Federal de Lavras (UFLA), Lavras, Minas Gerais, Brazil; 3 Lancaster Environment Centre, Lancaster University, Lancaster, Lancashire, United Kingdom; Universidade Federal de Vicosa, BRAZIL

## Abstract

Arthropod community composition in agricultural landscapes is dependent on habitat characteristics, such as plant composition, landscape homogeneity and the presence of key resources, which are usually absent in monocultures. Manipulating agroecosystems through the insertion of in-field floral resources is a useful technique to reduce the deleterious effects of habitat simplification. Food web analysis can clarify how the community reacts to the presence of floral resources which favour ecosystem services such as biological control of pest species. Here, we reported quantitative and qualitative alterations in arthropod food web complexity due to the presence of floral resources from the Mexican marigold (*Tagetes erecta* L.) in a field scale lettuce community network. The presence of marigold flowers in the field successfully increased richness, body size, and the numerical and biomass abundance of natural enemies in the lettuce arthropod community, which affected the number of links, vulnerability, generality, omnivory rate and food chain length in the community, which are key factors for the stability of relationships between species. Our results reinforce the notion that diversification through insertion of floral resources may assist in preventing pest outbreaks in agroecosystems. This community approach to arthropod interactions in agricultural landscapes can be used in the future to predict the effect of different management practices in the food web to contribute with a more sustainable management of arthropod pest species.

## Introduction

In agricultural landscapes richness and abundance of arthropods are dependent on local habitat characteristics, which are determined by the different strategies used in the management of the agroecosystem [1, 2]. Several pieces of evidence indicate that arthropod communities are directly affected by field simplification and loss of non-crop habitats in monocultures, causing distortion in species relative abundance, removing natural enemies from the crop may increase the likelihood for outbreaks [3–5]. The absence of key resources, such as shelter, alternative food (prey, hosts and nectar) and alternative habitat, is the most important factor limiting the effectiveness of ecosystem services, such as the biological control of arthropod pest species in monocultures [6]. Habitat manipulation through the insertion of flower strips adjacent to or within crops is an useful technique to counteract landscape simplification, and to provide resources for service-providers [7].

Crop diversification increases species richness, abundance and fitness of natural enemies, which can minimize pest damage and increases yield in commercial crops [8–10]. However, resource diversification studies usually focus only on natural enemies and how species abundance and richness are affected by attractiveness, phenology, floral accessibility, seeds, pollen and nectar production of non-crop plants [11–16]. Notwithstanding, diversity indices, richness and abundance are not sufficient to describe changes in community structure, mainly by ignoring interaction between species [17]. Moreover, these descriptors are superficial and may be of little use describing plant/crop outcome, ecosystem functioning and provision of essential services such as the biological control of arthropod pest species [18, 19].

Food web analysis provides an alternative approach and constitutes a powerful mechanism for quantifying species interactions at different trophic levels in a community and revealing functional biodiversity components in an ecological network [20, 21]. This representative approach allows analysis of similarities between complex systems originated from different environments [22, 23], providing robust information about community structure and insights into the dynamic processes associated with their structuring, including robustness to species loss, stability of herbivore species population and susceptibility to species invasion [21, 24–27].

Furthermore, fine and more comprehensive details about ecological community descriptors can be visualized using a food web trivariate approach, combining data from numerical abundance, body size and biomass abundance [28]. Overviews of literature reports dealing with flower resources and biological control enhancements do not describe or confirm alteration in species interaction, energy flow and changes in trophic webs levels. The quantification of relationships between plants and arthropod species through food webs can clarify the ecological mechanisms favouring the biological control of pest species in diversified environments. Here, in addition to resulting in higher abundance and richness, it was hypothesized that presence of floral resources also modifies community dynamics in a horticultural commercial sized crop, based on a smallholder organic model farm, optimizing provision of essential ecosystem services such as biological control, which can result in a more stable agroecosystem. Thus, the objective of this study was to evaluate how food web complexity is affected by the presence of additional floral resources in agroecosystem community networks.

## Material and methods

### Ethic statement

This study did not involve any endangered or protected species and no specific permission was required.

### Experimental site

**Fig 1:**
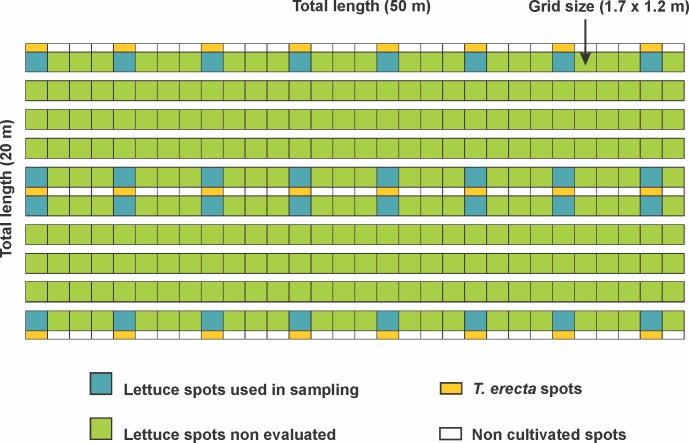
Design of the field experiment representing grids of lettuce and the flower resource Mexican marigold (*T*. *erecta*).

The study was carried out at the Horticultural Experimental Station of the Federal University of Lavras (Lavras, Minas Gerais, Brazil; 21°13'51.06"S, 44°58'34.36” W) from September/2012 to January/2013, representing a smallholder organic model farm, with 2 ha area with cultivated with diversified vegetables crops (e.g., tomato, cabbage, kale, aromatic plants). The field experiments were performed in a 0.1 ha area (20 m width and 50 m length), representing a commercial sized crop, isolated from the native vegetation by a 2 m border maintained without plants. The cultivated area was divided in beds (1.2 m width, 45 m length) spaced 0.5 m from one another composed by approximately 30 grids (1.7 m width, 1.2 m length) (Fig 1).

Lettuce (*Lactuca sativa* var. Solaris) was used as a model vegetable crop, due to its low defense against herbivores during commercial stage. Seeds were obtained from Seminis© (Curitiba, PR, Brazil) and seedlings were cultivated in nursery for 30 days before transplantation to the field, in which lettuce plants were cultivated in four lines per bed using 0.3 x 0.35 m spacing.

Plants of the Mexican marigold *Tagetes erecta* L. were used as a model floral resource due to its capacity to attract and sustain natural enemies in agroecosystems [29]. Mexican marigold seeds were obtained from ISLA© Sementes (Porto Alegre, RS, Brazil) and seedlings were cultivated in nursery for 20 days before transplant to the field. They were cultivated as a floral resource in the border and between the beds of the vegetable crop field in small linear plots with 1.7 m and plant spacing of 0.15 m; within each bed, the plots were 5.1 m apart from each other (Fig 1).

Lettuce plants were cultivated in three consecutive commercial cycles (35 days each) alongside floral resource plots with *T*. *erecta* plants (which were not changed during the three lettuce cycles) allowing evaluation of possible alterations in food web composition with development stage of the *T*. *erecta*, which was classified as either: 1) pre-flowering, encompassing the period between seedling transplant into the field, 30 days after germination, until the appearing of the floral buds; 2) complete flowering, containing all plants with flowers, immature seeds and few floral buds; and 3) late-flowering, with the majority of the plants exhibiting flowers with mature seeds, few flowers with immature seeds and almost no floral buds.

### Arthropod and plant sampling

Arthropod and lettuce plant sampling were performed 15, 21, 28 and 35 days after seedling transplant to the crop field in all the three crop cycles, in six randomly selected grids per week. The average of three plants per grid constituted a replicate, totalling 18 lettuce per week. During the process, the entire plants were covered by a translucent plastic bag and totally removed from the field. Care was taken to avoid contact with the plant before covering to allow collection of fast moving arthropods.

Fresh biomasses of lettuce plants were determined with an analytical balance (Shimadzu AUW220D, Kyoto, Japan), aiming to measure the relationship between floral resources and biological control in the agroecosystem. The arthropods from each sampled lettuce were subsequently identified and counted to obtain species richness (*S*) and numerical abundance (*N*; individuals/m^2^). Specific body mass (*M*; mg) was obtained by the average of weighing 10 arthropods from each species on an electronic scale (model XS3DU, Mettler Toledo, Columbus, OH, EUA). Aiming to analyze energy flow patterns in the community [28], biomass abundance (*B*; mg/m^2^) was calculated, by multiplying numerical abundance per plant by specific body mass (i.e., *B* = *N* x *M*).

The collected specimens were maintained in vials with 70% alcohol until the identification process, which was performed by trained personnel using available taxonomic keys and reference collections from the Entomology Museum of the Federal University of Lavras (S1 Table).

The average number of *T*. *erecta* flowers per plot/week was determined by randomly sampling 8 floral resource plots per week.

### Food web connections

Interaction among species was first recognized by in-field visual observation. Specimens of arthropods were also collected and reared in laboratory, which permited the assessment of herbivory, parasitism, hyperparasitism and predatory behaviours [30, 31]. This step allowed to recognize approximately 82% of the trophic interaction observed (S2 Table). All relationships among the different arthropod species were confirmed by comparison with existing literature [32, 33]. Complementary interactions (19%) were computed based exclusively in the literature register. In a food web with cannibalism cycles (A eats A) and mutual predation loops (A eats B eats A, or longer) a species may have different trophic positions, depending on which food chain is specified. As stated in the literature, cannibalism cycles and mutual predation loops were ignored in computing trophic levels, to assure that they are all finite, since loops makes it impossible to delimit the food web [28].

### Data analyses

Data from species richness (*S*), numerical abundance (*N*), body mass (*M*), biomass abundance (*B*) and all connections registered between arthropods (S2 and S3 Tables) were used for the food web parameters computation (Table 1) [28, 34, 35]. All parameters were calculated in R [36] using the cheddar package [37].

**Table 1:** Food web parameters evaluated in the experiment.

Parameter	Definition
**Species properties**	
Number of nodes	Species richness in the food web (*S*)
Proportion of top, intermediate and basal taxa	Percentage of species in each trophic height
Ratio of prey to consumers	Relationship between preys and consumers
**Link properties**	
Number of trophic links	Number of trophic links among all species in the food web (*L*)
Link density	Exhibits scale invariance or weak dependence on food-web size
Connectance	Number of possible links over possible links in a community (*L/S*^2^)
Average link length	Average of link lenght in the food web
Proportion of links between top, intermediate and basal taxa	Estimate percentage of links among species in different status (basal, top and intermediate)
**Chain properties**	
Average chain length	Is defined as the number of links running from a top predator to a basal species
**Omnivory properties**	
Degree of omnivory	Percentage of taxa that consuume prey from more than one trophic level
**Consumer-prey asymmetries**	
Generality	Mean number of prey per consumer
Vulnerability	Mean number of consumers per prey

Trophic webs from pre-flowering, flowering and late flowering period were plotted using the trivariate pattern *N*, *M* and *B*, which were log-transformed with base 10 to reduce the dimensionality of the data [28]. Trends of *N*, *M* and *B* distribution over time for each trophic level were analyzed using multiple linear regressions. The curve-fitting software TableCurve 3D (Systat, San Jose, CA, USA) was used to estimate the best fit of the equation. Low error, high F-value and R^2^ were the parameters used for model selection and validation. In addition, linear regression was used to assess the effect of the number of *T*. *erecta* flowers on species richness, numerical abundance and biomass abundance for each trophic level.

## Results

### Species richness

**Fig 2:**
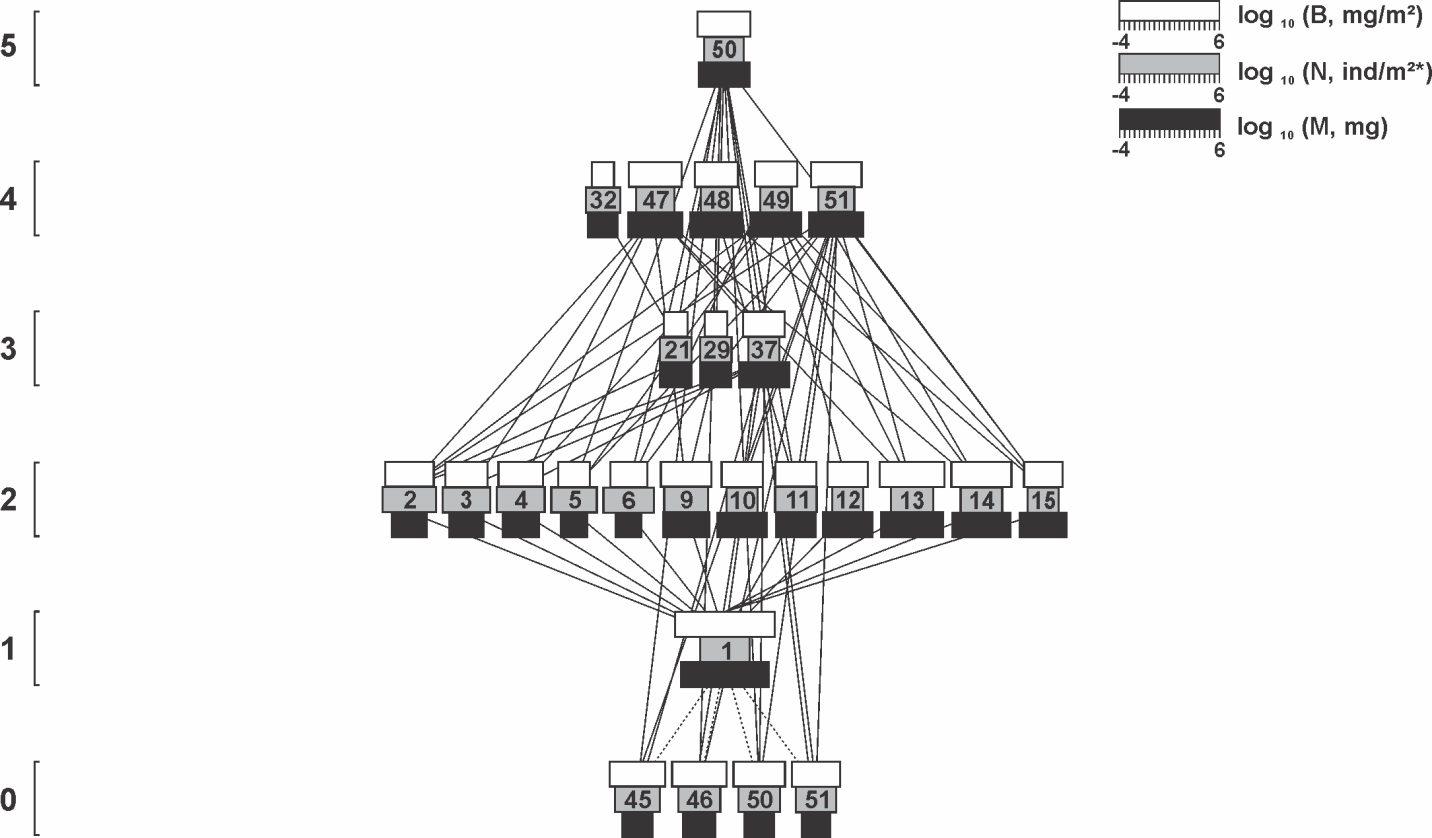
The arthropod food web associated with lettuce plants during the pre-flowering stage of *T*. *erecta*. The width of the black, gray and white horizontal bars shows log_10_ body mass (mg), log_10_ numerical abundance (individuals/m^2^) and log_10_ biomass abundance (mg/m^2^). Solid lines represent direct consumption interaction. Dotted line represents presence, but without direct consumption of living tissues. Species numbers are identified in Table 2. The vertical position indicates trophic level. Horizontal position is arbitrary. Isolated species, cannibalism or loops are ignored.

**Fig 3:**
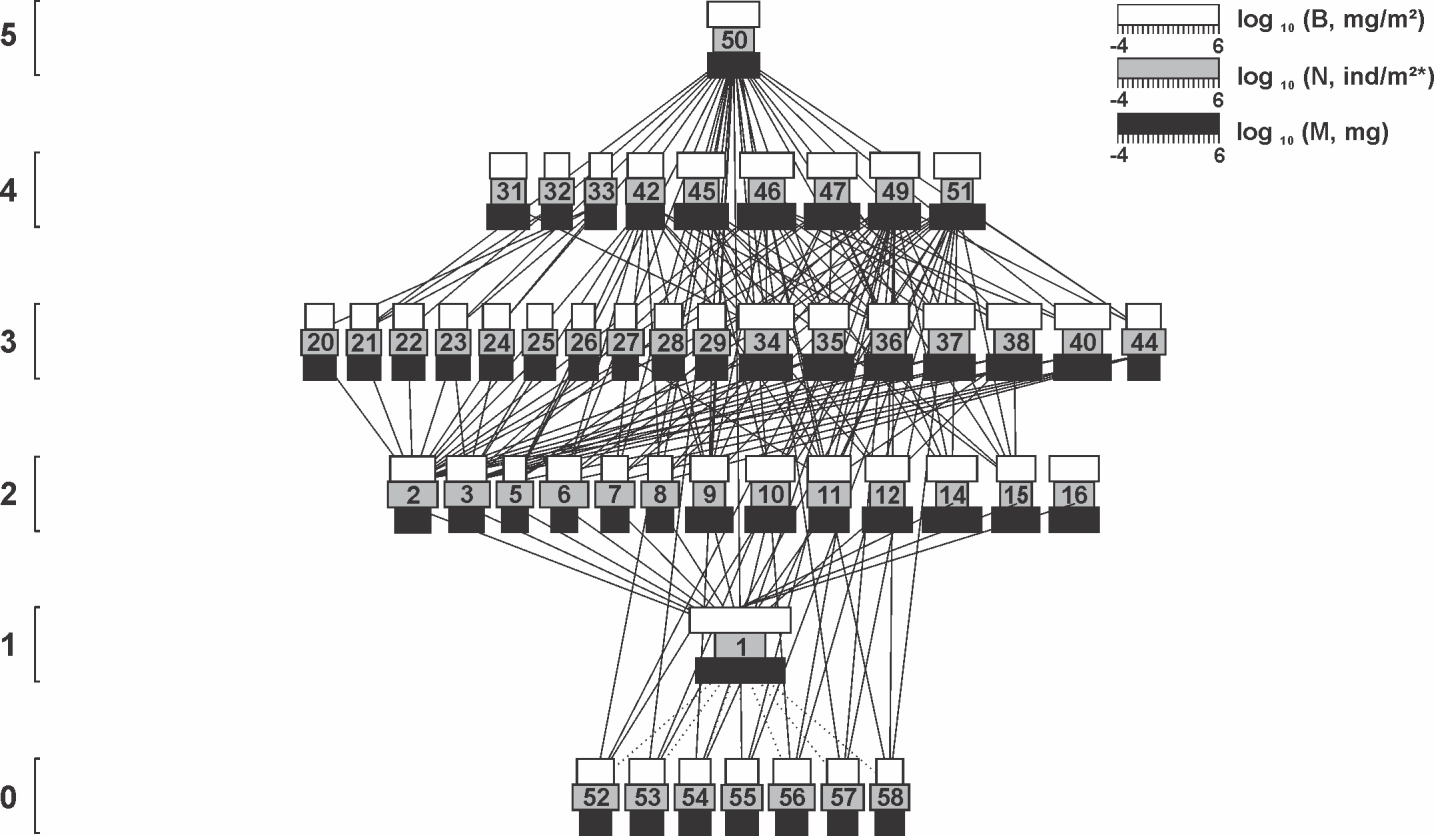
The arthropod food web associated with lettuce plants during the flowering stage of *T*. *erecta*. The width of the black, gray and white horizontal bars shows log_10_ body mass (mg), log_10_ numerical abundance (individuals/m^2^) and log_10_ biomass abundance (mg/m^2^). Solid lines represent direct consumption interaction. Dotted line represents presence, but without direct consumption of living tissues. Species numbers are identified in Table 2. The vertical position indicates trophic level. Horizontal position is arbitrary. Isolated species, cannibalism or loops are ignored.

**Fig 4:**
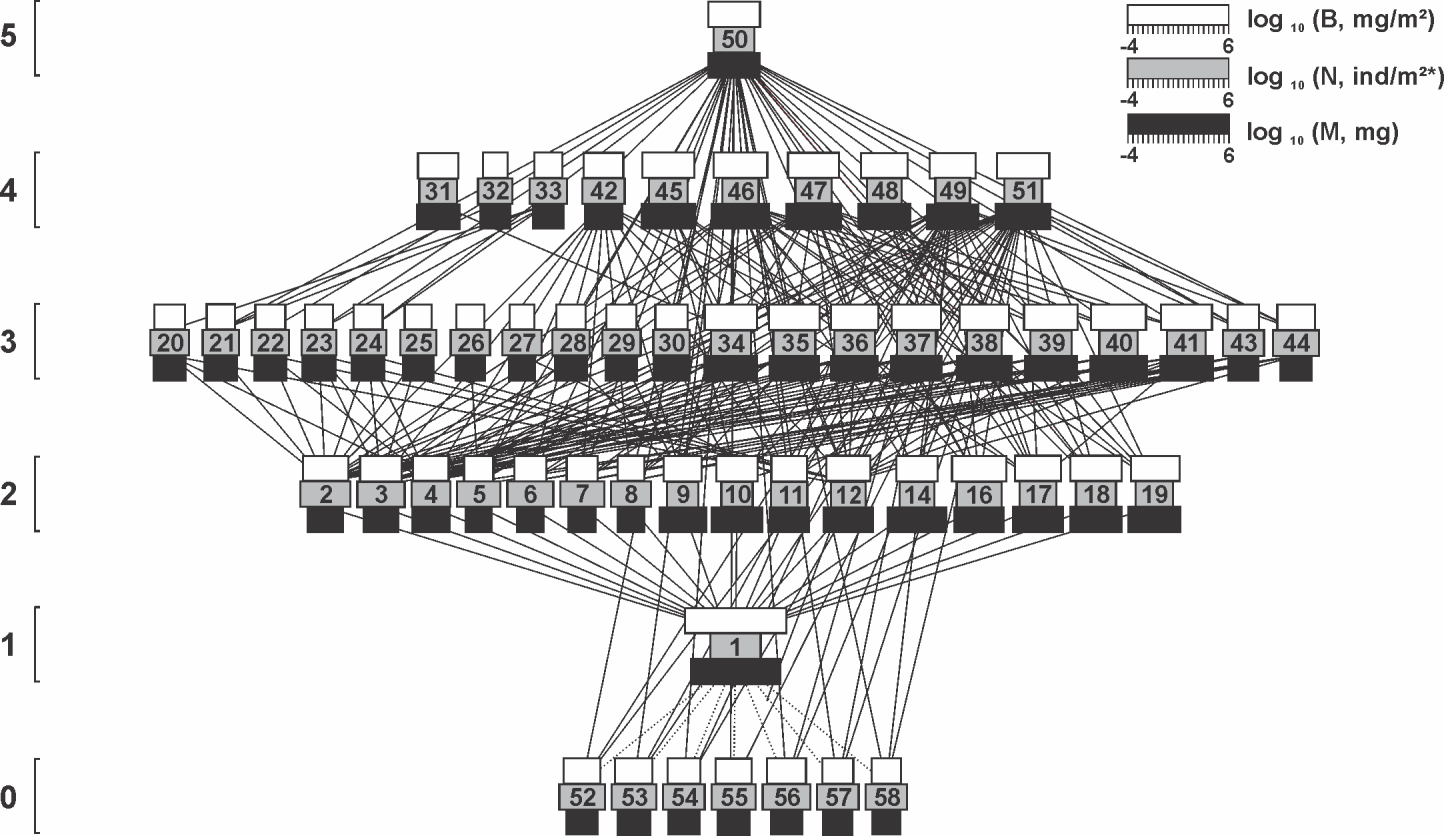
The arthropod food web associated with lettuce plants during the late flowering stage of *T*. *erecta*. The width of the black, gray and white horizontal bars shows log_10_ body mass (mg), log_10_ numerical abundance (individuals/m^2^) and log_10_ biomass abundance (mg/m^2^). Solid lines represent direct consumption interaction. Dotted line represents presence, but without direct consumption of living tissues. Species numbers are identified in Table 2. The vertical position indicates trophic level. Horizontal position is arbitrary. Isolated species, cannibalism or loops are ignored.

A total of 57 arthropod taxa were collected throughout the three lettuce cultivation cycles and assembled in food webs (Figs 2, 3 and 4; Table 2). Remarkable differences in species richness were registered during different floral resource developmental stages. In the pre-flowering cultivation, the entire food web was composed by 27 taxa, increasing to 49 and 56 during flowering and late flowering respectively (Table 2). Linear regression indicated a direct relationship between alterations in richness and the increasing number of *T*. *erecta* flowers in the field (y = 0.94 + 0.16x; R^2^ = 0.39; F = 46.09; P < 0.001). Although the difference in richness was significant, the arthropods collected were distributed across the 5 trophic levels with at least one representative of each level in each the three cultivations (Figs 2, 3 and 4).

**Table 2:** Average body mass (*M;* mg), numerical abundance (*N;* individuals/m^2^ of crop), biomass abundance (*B;* mg/m^2^ of crop) and connectivity status of arthropods registered in lettuce plants during different development stages of the floral resource *Tagetes erecta*.

			Food web		Pre flowering		Flowering		Late Flowerting	
Species		Taxonomy	status	M	N	B	N	B	N	B
**Producer**		** **	** **	** **	** **	** **	** **	** **	** **	** **
1	*Lettuce*	* *	Producer		9.42	616882	9.42	644061	9.42	702269
**Herbivores**		** **								
2	*Myzus persicae*	*Hemiptera*	Herbivore	0.360	20.933	7.536	8.243	2.967	8.766	3.156
3	*Aulacorthum solani ala*	*Hemiptera*	Herbivore	0.280	5.757	1.612	3.271	0.916	4.841	1.355
4	*Uroleucon ambrosiae*	*Hemiptera*	Herbivore	0.500	6.018	3.009	―	―	0.654	0.327
5	*Frankliniella schultzei*	*Thysanoptera*	Herbivore	0.044	3.663	0.161	0.393	0.017	1.832	0.081
6	*Caliothrips phaseoli*	*Thysanoptera*	Herbivore	0.042	11.644	0.489	5.888	0.247	3.663	0.154
7	*Neohydatothrips gracilipes*	*Thysanoptera*	Herbivore	0.052	―	―	0.785	0.041	2.224	0.116
8	*Echinothrips mexicanus*	*Thysanoptera*	Herbivore	0.048	―	―	0.523	0.025	0.916	0.044
9	*Liriomyza trifolii*	*Diptera*	Herbivore	4.430	2.486	11.012	0.131	0.580	0.131	0.579
10	*Diabrotica speciosa*	*Coleoptera*	Herbivore	10.191	0.131	1.333	0.654	6.667	0.131	1.333
11	*Empoasca kraemeri*	*Hemiptera*	Herbivore	0.900	1.439	1.295	1.963	1.766	0.393	0.353
12	*Sonesimia grossa*	*Hemiptera*	Herbivore	8.564	0.131	1.120	0.262	2.241	0.131	1.120
13	*Naupactus rivulosus*	*Coleoptera*	Herbivore	175.480	1.832	321.421	―	―	―	―
14	*Lagria villosa*	*Coleoptera*	Herbivore	71.778	1.439	103.301	0.131	9.391	0.262	18.782
15	*Ferrariana trivitata*	*Hemiptera*	Herbivore	5.264	0.131	0.689	0.131	0.689	―	―
16	*Xyonizius californicus*	*Hemiptera*	Herbivore	8.291	―	―	0.915	7.586	2.748	22.780
17	*Hortensia similis*	*Hemiptera*	Herbivore	9.830	―	―	―	―	0.393	3.858
18	*Isotes bertonii*	*Coleoptera*	Herbivore	14.022	―	―	―	―	0.916	12.842
19	*Sternocolaspis quatuordeimcosta*	*Coleoptera*	Herbivore	13.826	―	―	―	―	0.523	7.236
**Parasitoids**		** **								
20	*Aphidius colemani*	*Hymenoptera*	Specialist	0.167			0.393	0.066	0.654	0.109
21	*Diaeretiella rapae*	*Hymenoptera*	Specialist	0.172	0.131	0.023	0.262	0.045	0.393	0.068
22	*Lysiphebus testaceipes*	*Hymenoptera*	Specialist	0.179	―	―	0.523	0.094	0.523	0.094
23	*Praon volucre*	*Hymenoptera*	Specialist	0.206	―	―	0.262	0.054	0.262	0.054
24	*Aphidius ervi*	*Hymenoptera*	Specialist	0.189	―	―	0.262	0.049	0.393	0.074
25	*Aphelinus asychis*	*Hymenoptera*	Specialist	0.122	―	―	0.262	0.032	0.393	0.048
26	*Ceranisus menes*	*Hymenoptera*	Specialist	0.077	―	―	0.262	0.020	0.785	0.060
27	*Anagrus empoascae*	*Hymenoptera*	Specialist	0.036	―	―	0.393	0.014	0.785	0.028
28	*Chrysocharis vonones*	*Hymenoptera*	Specialist	0.142	―	―	0.393	0.056	0.523	0.074
29	*Opius dissitus*	*Hymenoptera*	Specialist	0.139	0.131	0.018	0.393	0.055	0.654	0.091
30	*Centistes gasseni*	*Hymenoptera*	Specialist	0.409	―	―	―	―	0.393	0.161
31	*Diplazon laetatorius*	*Hymenoptera*	Specialist	1.788	―	―	0.262	0.468	0.523	0.936
32	*Alloxysta fuscicornis*	*Hymenoptera*	Specialist	0.103	―	―	0.262	0.027	0.262	0.027
33	*Alloxysta victrix*	*Hymenoptera*	Specialist	0.118	0.262	0.015	0.131	0.015	0.523	0.062
**Predators**		** **								
34	*Toxomerus procrastinatus*	*Diptera*	Specialist	16.164	―	―	1.832	29.607	0.916	14.804
35	*Condylostylus erectus*	*Diptera*	Specialist	7.463	―	―	0.131	0.976	1.439	10.741
36	*Aphidoletes sp*	*Diptera*	Specialist	4.750	―	―	0.262	1.243	1.047	4.972
37	*Eriopsis conexa*	*Coleoptera*	Specialist	11.530	0.131	1.509	1.047	12.068	0.654	7.543
38	*Cycloneda sanguinea*	*Coleoptera*	Specialist	29.880	―	―	0.785	23.456	0.262	7.819
39	*Harpasus eversmanni*	*Coleoptera*	Specialist	15.880	―	―	―	―	1.308	20.776
40	*Harmonia axyridis*	*Coleoptera*	Specialist	41.470	―	―	0.785	32.554	0.654	27.128
41	*Hippodamia convergens*	*Coleoptera*	Specialist	17.320	―	―	―	―	0.654	11.330
42	*Orius insidiosus*	*Hemiptera*	Generalist	0.540	―	―	0.654	0.353	1.570	0.848
43	*Stomatothrips angustipennis*	*Thysanoptera*	Specialist	0.113	―	―	―	―	1.178	0.133
44	*Franklinothrips vespiformis*	*Thysanoptera*	Specialist	0.142	―	―	1.832	0.260	3.794	0.539
45	*Polybia paulista*	*Hymenoptera*	Generalist	21.967	―	―	0.262	5.748	0.654	14.370
46	*Euborellia annulipes*	*Dermaptera*	Generalist	52.709	―	―	0.393	20.688	0.393	20.688
47	*Doru luteipes*	*Dermaptera*	Generalist	28.643	0.654	18.737	0.262	7.495	0.393	11.242
48	*Cheiracanthium inclusum*	*Araneae*	Generalist	16.560	0.131	2.167	―	―	0.262	4.333
49	*Hasarius adansoni*	*Araneae*	Generalist	13.165	0.131	1.722	0.523	6.890	0.262	3.445
50	*Oxyopes salticus*	*Araneae*	Generalist	12.665	0.916	11.599	1.047	13.256	1.047	13.256
51	*Menemerus bivittatus*	*Araneae*	Generalist	27.860	0.393	10.935	0.131	3.645	0.523	14.580
**Detritivorous**		** **								
52	*Sminthurus rosai*	*Collembola*	Detritivore	0.126	2.093	0.264	3.663	0.462	3.402	0.429
53	*Tullbergia minensis*	*Collembola*	Detritivore	0.181	1.047	0.189	1.701	0.308	2.748	0.497
54	*Lepidocyrtus pallidus*	*Collembola*	Detritivore	0.138	―	―	0.916	0.126	1.308	0.181
55	*Entomobrya ataquensis*	*Collembola*	Detritivore	0.164	―	―	1.047	0.172	2.224	0.365
56	*Seira sp*	*Collembola*	Detritivore	0.142	―	―	3.533	0.502	5.233	0.743
57	*Arlea lucifuga*	*Collembola*	Detritivore	0.092	1.047	0.096	1.439	0.132	1.308	0.120
58	*Sphaeridia biniserata*	*Collembola*	Detritivore	0.066	1.178	0.078	0.654	0.043	1.308	0.086

### Food web properties

The increasing richness, probably triggered by the presence of floral resources, also affected the number of trophic links during the flower and late flowering cultivation periods, which were two and three-times greater, respectively, when compared with pre-flowering. Link density, which is directly dependent on link number and richness, also followed the same ascending pattern as the Mexican marigold developed (2.778, 3.878 and 4.578 in pre-flowering, flowering and late flowering stages respectively), (Table 3).

**Table 3:** Values of food web descriptors for the arthropod food web associated with lettuce plants during the three development stages of the floral resource *Tagete erecta*.

	Pre-flowering	Flowering	Late flowering
Species properties			
Number of nodes	27	49	57
Proportion of top taxa	0.222	0.245	0.232
Proportion of intermediate taxa	0.704	0.714	0.737
Proportion of basal taxa	0.074	0.041	0.035
Ratio of prey to consumers	0.840	0.787	0.800
Link properties			
Number of trophic links	75	190	261
Link density	2.778	3.878	4.578
Connectance	0.103	0.079	0.080
Average link length	2.776	2.174	2.025
Proportion of links between			
Top and intermediate	0.667	0.537	0.503
Intermediate and intermediate	0.120	0.358	0.409
Intermediate and basal	0.213	0.105	0.088
Chain properties			
Average chain length	2.274	2.750	2.787
Standard deviation of chain length	0.450	0.434	0.409
Omnivory properties			
Degree of omnivory	0.074	0.122	0.122
Consumer-prey asymmetries			
Generality	3.000	4.043	4.745
Vulnerability	3.571	5.135	5.931
SD of standardised generality	1.469	1.496	1.474
SD of standardised vulnerability	0.867	0.964	0.988

The proportions of taxa in each food web status (producer, consumer) varied during experiment (Table 3). Producer (lettuce plants) represented 7% of all taxa the in the pre-flowering cultivation, decreasing to 4 and 3% in flowering and late flowering cultivation periods respectively. In contrast, the proportion of intermediate taxa rose from 70% in pre-flowering to 73% in late flowering, due to additions of parasitoids and predators in this category, which were also responsible for lower prey-to-consumer ratios in flowering and late flowering treatments (Table 3). The insertion of intermediate taxa affected the probability of any two species interacting with each other (see connectance in Table 3) shortening the average distance from resource to consumers (see average link length Table 3). However, this compartmentalization resulted in an ascending average chain length from pre-flowering to late flowering cultivation (Table 3).

The additions of detritivores, herbivores, predators and parasitoids changed not only community composition, but also the asymmetries between prey and consumers during the experiment (Figs 2, 3 and 4). As an example, generality was affected by the development of flowers in the marigold *T*. *erecta*, increasing from 3.00 during the pre-flowering cultivation, to 4.04 during flowering and 4.75 in late flowering (Table 3). The same pattern was registered for the food web vulnerability, which responded to augmentative number of flowers in the field increasing by 60% its value between the pre-flowering (3.57) and late flowering cultivation (5.93) (Table 3).

### Numerical abundance

The total food web numerical abundance, including all arthropods collected, was also altered by the floral resource developmental stages, responding positively to the number of flowers per plot (y = 4.05 + 1.53x; R^2^ = 0.51; F = 73.08; *P* < 0.001). However, the same pattern was not registered in all trophic levels. The abundance of natural enemies located on the third (y = 5.35 + 1.24x; R^2^ = 0.63; F = 120.37; *P* < 0.001) and fourth trophic levels (y = 4.21 + 0.30x; R^2^ = 0.24; F = 17.96; *P* < 0.001), in addition to detritivores (trophic level 0) (y = 20.14 + 0.95x; R^2^ = 0.57; F = 96.15; *P* < 0.001), positively responded to the presence of flowers in the field. Inversely to the natural enemies, herbivores located in the second trophic level significantly decreased in abundance as marigold flowering advanced (y = 140.41–1.5x; R^2^ = 0.13; F = 9.83; *P* = 0.002). The responses of some specific arthropod groups deserve attention, such as the aphids, whose abundance also responded negatively to the number of *T*. *erecta* flowers in the field (y = 1.33–0.51x; R^2^ = 0.29; F = 29.21; *P <* 0.001), decreasing during flowering and late flowering periods, probably due to the increasing number of natural enemies. Another interesting response was registered for thrips (Thysanoptera), encompassing two of the agricultural pest species collected during the study, *Caliothrips phaseoli* Hood, 1912 and *Frankliniella schultzei schultzei* (Trybom, 1910) (Thripidae), which significantly decreased with the presence of flowers in the field (y = 0.6–0.22x; R^2^ = 0.26; F = 25.46; *P <* 0.001). Conversely, harmless thrips species such as *Echinothrips mexicanus* Moulton, 1911 and *Neohydatothrips gracilipes* Hood, 1924 (Thripidae), which were sampled in lettuce plants, increased their numerical abundance during the experiment (y = - 0.005–0.102x; R^2^ = 0.30; F = 31.24; *P <* 0.001), denoting a specific substitution triggered by floral resources.

**Fig 5:**
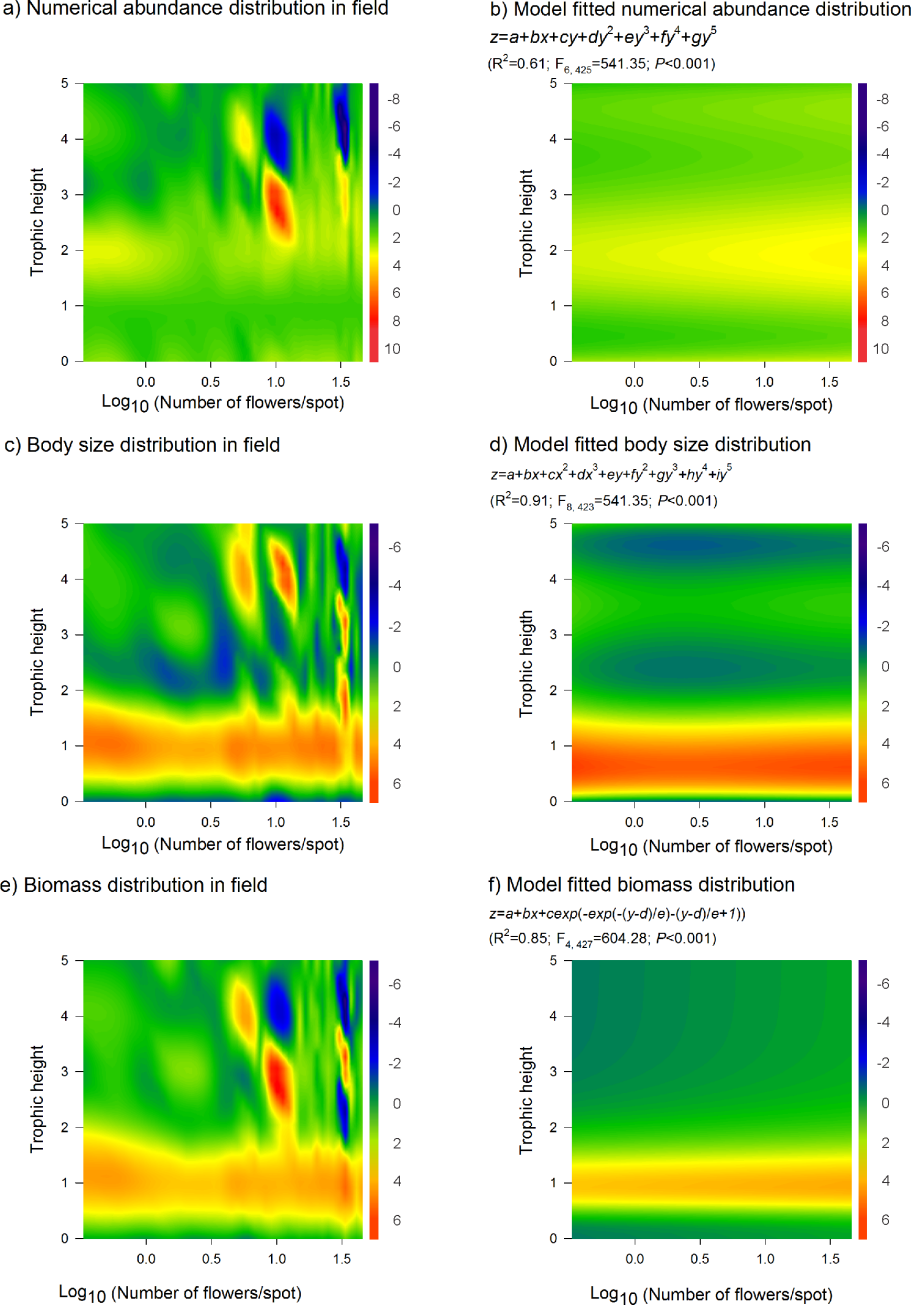
Distribution of empirical and simulated numerical abundance (individuals/m^2^), body mass (mg) and biomass abundance (mg/m^2^) through trophic height in function of the number of *T*. *erecta* flowers in field (number of flowers/spot). The color-graded scale at the right of each plot represents the level of either abundance (a,b), body size (c,d), or biomass (e,f) distribution of the lettuce-associated arthropods. The lowest to the highest levels are represented by the transition from dark blue to dark red respectively.

The observed data were subsequently subjected to multiple regression analysis and a polynomial model equation (*z* = *a* + *bx* + *cy* + *dy*^2^ + *ey*^3^ + *fy*^4^ + *gy*^5^) was obtained to explain the dispersion of numerical abundance in the different trophic levels mediated by the increasing number of marigold flowers (R^2^ = 0.60; F_6, 425_ = 107.25; *P* < 0.001) (Fig 5A) and used to generate the simulated distribution (Fig 5B). Numerical abundance increased in response to a higher number of flowers (Fig 5A).

### Body mass

Total body mass was not affected by the addition of floral resources in the field. However, specific effects were noticed in particular trophic levels. Average body mass of herbivores decreased during the marigold flowering (y = 10.71–6.14x; R^2^ = 0.23; F = 21.99; *P* < 0.001). In contrast, specialist natural enemies located in third trophic level increased their average body mass with the presence of floral resources in the field (*y* = - 6.14*x* + 10.71; R^2^ = 0.23; F = 21.99; *P* < 0.001). A more complex *X-Y* polynomial model (*z* = *a* + *bx* + *cx*^2^ + *dx*^3^ + *ey* + *fy*^2^ + *gy*^3^ + *hy*^4^ + *iy*^5^) better fitted the body mass distribution in different trophic levels during the marigold flowering (R^2^ = 0.91; F_8, 423_ = 541.35; *P* < 0.001) (Fig 5C). The model was used to simulate the distribution pattern (Fig 5D). In both graphics were registered increasing body sizes in higher trophic levels.

### Biomass abundance

Total biomass increased due to presence of flower resources in the field (y = 0.58 + 0.14x; R^2^ = 0.44; F = 55.67; p > 0.001). However, change in total biomass reflected the increasing biomass in specific trophic levels, particularly of detritivores (y = -0.13 + 0.51x; R^2^ = 0.70; F = 168.82; *P* < 0.001) and natural enemies located in third trophic level (y = 0.37 + 0.92x; R^2^ = 0.45; F = 58.5; *P* < 0.001). Following the same pattern registered for numerical abundance, aphid biomass decreased with the presence of higher number of flowers per plot (y = 0.86–0.44x; R^2^ = 0.50; F = 71.98; *P* < 0.001). Data was assessed by multiple regression analysis and the non-linear extreme value model equation (z = a + bx + cexp(−exp(−(y−d)/e)−(y−d)/e + 1)) was selected to explain the dispersion of biomass abundance in the five trophic levels mediated by increasing flower number during the three field cultivations (R^2^ = 0.85; F_4, 427_ = 604.28; *P* < 0.001) (Fig 5E). The model was used to access the simulated distribution (Fig 5F) showing translocation of biomass from basal to higher trophic levels responding to a high number of flowers in field.

## Discussion

Planting of *T*. *erecta* within the crop modified the arthropod community associated with lettuce plants, changing the structure, biomass distribution, assumed species interactions and consumer/prey properties in the arthropod food webs. However, identifying the mechanisms involved in these changes is complex and requires a comprehensive approach [38]. In the first scenario, the presence of non-crop plants in the agroecosystem likely diversified microhabitats, allows the recruitment and conservation of species with different dietary, ecological and habitat requirements mainly due to the increasing availability of floral resources, which results in a diversified community [39–41]. The increasing complexity in the lettuce crop, arising from flowers, nectar and shelter, partially explains the higher richness and abundance in lettuce recorded during the marigold flowering stages [42]. The main trophic levels responsible for species richness increase were detritivores (ranked as trophic level 0), composed principally of springtails; and natural enemies, either specialists (third trophic level) or generalists (fourth trophic level), whose positive responses to the increasing complexity of the environment have been previously reported [43, 44].

Secondly, the availability of alternative food resources might also explain the observed changes in arthropod community. Increased organic matter arising from plant diversification probably benefited detritivorous arthropods [45]. As a third scenario, parasitoids and predators in the third trophic level were likely assisted directly or indirectly by the presence of non-prey food items in the marigold plants. Specialist natural enemies, mainly parasitoids, depend on these alternative food resources at least during part of their life cycle, and the absence of these resources, as during the marigold vegetative stage, has been shown to directly affect the abundance and richness of this group in the field [46, 47]. Finally, generalist natural enemies, especially spiders, are in a unique position in that they can potentially benefit from the three previous scenarios feeding on non-prey food from floral resources [12, 47], on specialist natural enemies, and on detritivores [45].

Higher richness of natural enemies consequently leads to higher number of trophic links in the food web, modifying the entire community structure and composition through a combination of bottom-up and top-down effects. The increasing number of links, sometimes with more than one prey per natural enemy species, may potentially reduce the abundance of harmful herbivores and their damage to the crop field [48, 49]. As an example, in this study there was a significant reduction in the abundance of herbivores, including arthropod pest species, during the marigold flowering stage. This reduction may be linked to the population increase of a second species (a detritivore, or herbivore present in the crop, or non-crop plant itself) mediating numerical increase of a third species located in a higher trophic level (generalist or specialist natural enemy). This process is known as "apparent competition" in which natural enemies shared by more than one prey species result in a negative interaction among the prey species [50].

Comparative analysis on the food web properties resulting from the different lettuce cultivations also demonstrates the community impact arising from the alteration of natural enemy numerical abundance. The susceptibility to predation in a food web can be measured using two parameters: generality (i.e. the average number of prey for natural enemy species), and vulnerability (i.e. the average number of consumers per prey). Increases of these two parameters as observed in these study, especially vulnerability, reflects changes in top-down control and predation strength, which results in large changes in biomass registered in the study, potentially reflecting the degree of energy propagation in the food web [51–54]. In addition to the presence/absence of natural enemies, another factor affecting the generality and vulnerability of the trophic network is the body size of consumers or prey, which may limit consumption in terrestrial environments [51]. In this study, during the Mexican marigold flowering stage, both the higher richness and abundance of natural enemies and the reduction of prey body size potentially were responsible for increasing community vulnerability and generality.

Curiously, connectance (*L/S*^2^) declined with increasing species richness, usually, food webs with low connectance are highly sensitive to species loss and this sensitivity tends to decrease with increasing connectance [55]. Theoretical research hypothesized that natural food webs in more complex environments are characterized by having a large number of weak interactions and a small numbers of strong interactions [56]. Therefore, rich food webs are more connected with weaker interactions between species on average, and this low strength is probably responsible for high stability even at low connectances [57, 58]. Thus, rich environments as observed during *T*. *erecta* flowering stages tend to be more robust to the loss of abundant less connected species and sensitive to loss of the rare more connected ones [22].

Omnivorism is another factor that can contribute to stability in diversified communities. Theoretical studies suggest that higher omnivory rates, as were observed during the flowering stage of marigold, might favour the stabilization of food webs with frequent, but weak, interactions [59, 60]. Empirical studies also suggest the stabilizing effect of omnivory in communities [61–63]. In the present study, increasing omnivorism was associated with the insertion of natural enemies at higher trophic levels of the food web. In general, environmental degradation causes a reduction in ecosystem size, especially through the loss of top predators, subsequently shortening the food chain length, as reported in the marigold vegetative stage [64, 65]. Food chain length is one of the community properties most affected by human activities [66, 67], and comparative study of observed food chain length originating from different lettuce cultivation allows us to hypothesize about the impact of the floral resources in decreasing impacts of agriculture management in the arthropod food web.

Earlier theoretical studies have demonstrated that longer food chain length tends to be less stable than shorter chain length [68–70]. However, recent studies have suggested that systems with long food chain length exhibit more stable population dynamics. The presence or absence of predatory species influences food web stabilization through its effects on herbivore populations [71], which are most affected by the addition of omnivorous species in the highest trophic levels [72]. In this study, the deleterious effect of diversification on the herbivore population was demonstrated by the systematic biomass loss as floral resources increased, which coincides with the longer food chain length and the higher rate of omnivorism registered. Lower stability between herbivore populations caused by natural enemy pressure was determined by the decline of dominant species, particularly important agricultural pests such as aphids. This disruption in dominance may also have allowed the recruitment and entrance of harmless herbivore species in the crop field.

The analyses of food web parameters allows us to hypothesize that agroecosystem diversification deeply affects the arthropod community dynamics, redirecting numerical abundance and biomass from herbivores to higher trophic levels, inducing a decrease in average body size of herbivores/natural enemies and potentially resulting in a more stable food web. Knowing the effect of additive floral resource on properties of crop food webs may allows optimization of floral resource use in integrated pest management programs by allowing the identification and matching of higher abundance and richness of beneficial arthropods with critical stages of the crop. As suggested in this study, the use of *T*. *erecta* plants as a floral resource during their flowering stage may prevent asynchronous colonization between natural enemies and pest population in field, considered the biggest challenge to effective natural biological control [73]. One limitation of this approach is related with the recognition of species feeding interaction in field. Next-generation sequencing is probably an important tool for these studies in the future, which is able to identify all DNA in digestive system of natural enemies and confirm interactions.

Our results suggested the addition of floral resources performs an effective and environmentally friendly strategy for farmers in the pest management. Food web complexity registered during flowering stages provided higher richness and abundance of specialists and generalists natural enemies in the crop, which increased community stability and functioning, benefiting biological control and avoiding outbreaks of herbivore population, such as aphids. Future studies should be conducted using other floral resources or different cultures to clarify the ecological relationships determining natural biological control. The effect of different external factors, such management practices, in the food web should also be tested in the future in order to contribute with a more sustainable management of harmful arthropods in the field.

## Supporting information

S1 Table Species and functional groups of arthropods associated with lettuce plants during the flowering stages of *T*. *erecta*. (PDF)

S2 Table Trophic links registered for the arthropods registered in the lettuce plants during the flowering stages of *T*. *erecta*. (PDF)

S3 Table Raw number of flowers, trophic level and number of individuals (m^2^) associated with lettuce plants during the experiment. (PDF)
