# Supplementary material for: Stability lies in flowers: Plant diversification mediating shifts in arthropod food webs
Source: PLoS One. 2018 Feb 16;13(2):e0193045. doi: 10.1371/journal.pone.0193045 (PMC5815608; doi:10.1371/journal.pone.0193045)
Supplement: S1 Table — (PDF) [file pone.0193045.s001.pdf]

**Taxon**	**Functional group**
Alloxysta fuscicornis	parasitoid
Alloxysta victrix	parasitoid
Anagrus empoascae	parasitoid
Aphelinus asychis	parasitoid
Aphidius colemani	parasitoid
Aphidius ervi	parasitoid
Aphidoletes sp	predator
Arlea lucifuga	decomposer
Aulacorthum solani	herbivore
Caliothrips phaseoli	herbivore
Centistes gasseni	parasitoid
Ceranisus menes	parasitoid
Cheiracanthium inclusum	predator
Chrysocharis vonones	parasitoid
Condylostylus erectus	predator
Cycloneda sanguinea	predator
Diabrotica speciosa	herbivore
Diaeretiella rapae	parasitoid
Diplazon laetatorius	parasitoid
Doru luteipes	predator
Echinothrips mexicanus	herbivore
Empoasca kraemeri	herbivore
Entomobrya ataquensis	decomposer
Eriopsis conexa	predator
Euborellia annulipes	predator
Ferrariana trivitata	herbivore
Frankliniella schultzei	herbivore
Franklinothrips vespiformis	predator
Harmonia axyridis	predator
Harpasus eversmanni	predator
Hasarius adansoni	predator
Hippodamia convergens	predator
Hortensia similis	herbivore
Isotes bertonii	herbivore
Lagria villosa	herbivore
Lepidocyrtus pallidus	decomposer
Lettuce	producer
Liriomyza trifolii	herbivore
Lysiphebus testaceipes	parasitoid
Menemerus bivittatus	predator
Myzus persicae	herbivore
Myzus persicae	herbivore

Taxon	Functional group
Myzus persicae	herbivore
Naupactus rivulosus	herbivore
Neohydatothrips gracilipes	herbivore
Opius dissitus	parasitoid
Orius insidiosus	predator
Oxyopes salticus	predator
Polybia paulista	predator
Praon volucre	parasitoid
Seira sp	decomposer
Sminthurus rosai	decomposer
Sonesimia grossa	herbivore
Sphaeridia biniserata	decomposer
Sternocolaspis quatuordeimcosta	herbivore
Stomatothrips angustipennis	predator
Toxomerus procrastinatus	predator
Tullbergia minensis	decomposer
Uroleucon ambrosiae	herbivore
Xyonizius californicus	herbivore
