# Supplementary material for: Stability lies in flowers: Plant diversification mediating shifts in arthropod food webs
Source: PLoS One. 2018 Feb 16;13(2):e0193045. doi: 10.1371/journal.pone.0193045 (PMC5815608; doi:10.1371/journal.pone.0193045)
Supplement: S2 Table — (PDF) [file pone.0193045.s002.pdf]

**Resource**	**Consumer**
Lettuce	*Myzus persicae*
Lettuce	*Aulacorthum solani*
Lettuce	*Uroleucon ambrosiae*
Lettuce	*Frankliniella schultzei*
Lettuce	*Caliothrips phaseoli*
Lettuce	*Neohydatothrips gracilipes*
Lettuce	*Echinothrips mexicanus*
Lettuce	*Liriomyza trifolii*
Lettuce	*Diabrotica speciosa*
Lettuce	*Empoasca kraemeri*
Lettuce	*Lagria villosa*
Lettuce	*Xyonizius californicus*
Lettuce	*Hortensia similis*
Lettuce	*Isotes bertonii*
Lettuce	*Sternocolaspis quatuordeimcosta*
Myzus persicae	*Aphidius colemani*
Uroleucon ambrosiae	*Aphidius colemani*
Myzus persicae	*Diaeretiella rapae*
Myzus persicae	*Lysiphebus testaceipes*
Uroleucon ambrosiae	*Lysiphebus testaceipes*
Myzus persicae	*Praon volucre*
Aulacorthum solani	*Praon volucre*
Uroleucon ambrosiae	*Praon volucre*
Myzus persicae	*Aphidius ervi*
Aulacorthum solani	*Aphidius ervi*
Uroleucon ambrosiae	*Aphidius ervi*
Myzus persicae	*Aphelinus asychis*
Aulacorthum solani	*Aphelinus asychis*
Frankliniella schultzei	*Ceranisus menes*
Empoasca kraemeri	*Anagrus empoascae*
Liriomyza trifolii	*Chrysocharis vonones*
Liriomyza trifolii	*Opius dissitus*
Diabrotica speciosa	*Centistes gasseni*
Toxomerus procrastinatus	*Diplazon laetatorius*
Aphidius ervi	*Alloxysta victrix*
Aphidius colemani	*Alloxysta victrix*
Praon volucre	*Alloxysta victrix*
Diaeretiella rapae	*Alloxysta victrix*
Diaeretiella rapae	*Alloxysta fuscicornis*
Myzus persicae	*Toxomerus procrastinatus*
Aulacorthum solani	*Toxomerus procrastinatus*
Uroleucon ambrosiae	*Toxomerus procrastinatus*

**Resource**	**Consumer**
Sminthurus rosai	*Toxomerus procrastinatus*
Tullbergia minensis	*Toxomerus procrastinatus*
Lepidocyrtus pallidus	*Toxomerus procrastinatus*
Entomobrya ataquensis	*Toxomerus procrastinatus*
Seira sp	*Toxomerus procrastinatus*
Arlea lucifuga	*Toxomerus procrastinatus*
Sphaeridia biniserata	*Toxomerus procrastinatus*
Myzus persicae	*Condylostylus erectus*
Aulacorthum solani	*Condylostylus erectus*
Uroleucon ambrosiae	*Condylostylus erectus*
Liriomyza trifolii	*Condylostylus erectus*
Myzus persicae	*Aphidoletes sp*
Aulacorthum solani	*Aphidoletes sp*
Uroleucon ambrosiae	*Aphidoletes sp*
Myzus persicae	*Eriopsis conexa*
Aulacorthum solani	*Eriopsis conexa*
Uroleucon ambrosiae	*Eriopsis conexa*
Sminthurus rosai	*Eriopsis conexa*
Tullbergia minensis	*Eriopsis conexa*
Lepidocyrtus pallidus	*Eriopsis conexa*
Entomobrya ataquensis	*Eriopsis conexa*
Seira sp	*Eriopsis conexa*
Arlea lucifuga	*Eriopsis conexa*
Sphaeridia biniserata	*Eriopsis conexa*
Myzus persicae	*Cycloneda sanguinea*
Aulacorthum solani	*Cycloneda sanguinea*
Uroleucon ambrosiae	*Cycloneda sanguinea*
Empoasca kraemeri	*Cycloneda sanguinea*
Sminthurus rosai	*Cycloneda sanguinea*
Tullbergia minensis	*Cycloneda sanguinea*
Lepidocyrtus pallidus	*Cycloneda sanguinea*
Entomobrya ataquensis	*Cycloneda sanguinea*
Seira sp	*Cycloneda sanguinea*
Arlea lucifuga	*Cycloneda sanguinea*
Sphaeridia biniserata	*Cycloneda sanguinea*
Hortensia similis	*Cycloneda sanguinea*
Myzus persicae	*Harpasus eversmanni*
Aulacorthum solani	*Harpasus eversmanni*
Uroleucon ambrosiae	*Harpasus eversmanni*
Sminthurus rosai	*Harpasus eversmanni*
Tullbergia minensis	*Harpasus eversmanni*
Lepidocyrtus pallidus	*Harpasus eversmanni*

**Resource**	**Consumer**
Entomobrya ataquensis	*Harpasus eversmanni*
Seira sp	*Harpasus eversmanni*
Arlea lucifuga	*Harpasus eversmanni*
Sphaeridia biniserata	*Harpasus eversmanni*
Myzus persicae	*Hippodamia convergens*
Aulacorthum solani	*Hippodamia convergens*
Uroleucon ambrosiae	*Hippodamia convergens*
Empoasca kraemeri	*Hippodamia convergens*
Sminthurus rosai	*Hippodamia convergens*
Tullbergia minensis	*Hippodamia convergens*
Lepidocyrtus pallidus	*Hippodamia convergens*
Entomobrya ataquensis	*Hippodamia convergens*
Seira sp	*Hippodamia convergens*
Arlea lucifuga	*Hippodamia convergens*
Sphaeridia biniserata	*Hippodamia convergens*
Hortensia similis	*Hippodamia convergens*
Frankliniella schultzei	*Orius insidiosus*
Caliothrips phaseoli	*Orius insidiosus*
Neohydatothrips gracilipes	*Orius insidiosus*
Echinothrips mexicanus	*Orius insidiosus*
Myzus persicae	*Orius insidiosus*
Aulacorthum solani	*Orius insidiosus*
Uroleucon ambrosiae	*Orius insidiosus*
Liriomyza trifolii	*Orius insidiosus*
Empoasca kraemeri	*Orius insidiosus*
Hortensia similis	*Orius insidiosus*
Myzus persicae	*Harmonia axyridis*
Aulacorthum solani	*Harmonia axyridis*
Uroleucon ambrosiae	*Harmonia axyridis*
Diabrotica speciosa	*Harmonia axyridis*
Sminthurus rosai	*Harmonia axyridis*
Tullbergia minensis	*Harmonia axyridis*
Lepidocyrtus pallidus	*Harmonia axyridis*
Entomobrya ataquensis	*Harmonia axyridis*
Seira sp	*Harmonia axyridis*
Arlea lucifuga	*Harmonia axyridis*
Sphaeridia biniserata	*Harmonia axyridis*
Frankliniella schultzei	*Stomatothrips angustipennis*
Caliothrips phaseoli	*Stomatothrips angustipennis*
Neohydatothrips gracilipes	*Stomatothrips angustipennis*
Echinothrips mexicanus	*Stomatothrips angustipennis*
Frankliniella schultzei	*Franklinothrips vespiformis*

**Resource**	**Consumer**
Caliothrips phaseoli	*Franklinothrips vespiformis*
Neohydatothrips gracilipes	*Franklinothrips vespiformis*
Echinothrips mexicanus	*Franklinothrips vespiformis*
Myzus persicae	*Doru luteipes*
Aulacorthum solani	*Doru luteipes*
Uroleucon ambrosiae	*Doru luteipes*
Liriomyza trifolii	*Doru luteipes*
Toxomerus procrastinatus	*Doru luteipes*
Condylostylus erectus	*Doru luteipes*
Aphidoletes sp	*Doru luteipes*
Eriopsis conexa	*Doru luteipes*
Cycloneda sanguinea	*Doru luteipes*
Harpasus eversmanni	*Doru luteipes*
Harmonia axyridis	*Doru luteipes*
Hippodamia convergens	*Doru luteipes*
Lagria villosa	*Doru luteipes*
Xyonizius californicus	*Doru luteipes*
Isotes bertonii	*Doru luteipes*
Sternocolaspis quatuordeimcosta	*Doru luteipes*
Myzus persicae	*Euborellia annulipes*
Aulacorthum solani	*Euborellia annulipes*
Uroleucon ambrosiae	*Euborellia annulipes*
Liriomyza trifolii	*Euborellia annulipes*
Toxomerus procrastinatus	*Euborellia annulipes*
Condylostylus erectus	*Euborellia annulipes*
Aphidoletes sp	*Euborellia annulipes*
Eriopsis conexa	*Euborellia annulipes*
Cycloneda sanguinea	*Euborellia annulipes*
Harpasus eversmanni	*Euborellia annulipes*
Harmonia axyridis	*Euborellia annulipes*
Hippodamia convergens	*Euborellia annulipes*
Lagria villosa	*Euborellia annulipes*
Xyonizius californicus	*Euborellia annulipes*
Isotes bertonii	*Euborellia annulipes*
Sternocolaspis quatuordeimcosta	*Euborellia annulipes*
Diaeretiella rapae	*Oxyopes salticus*
Lysiphebus testaceipes	*Oxyopes salticus*
Praon volucre	*Oxyopes salticus*
Aphidius ervi	*Oxyopes salticus*
Opius dissitus	*Oxyopes salticus*
Centistes gasseni	*Oxyopes salticus*
Chrysocharis vonones	*Oxyopes salticus*

Resource	Consumer
Frankliniella schultzei	*Oxyopes salticus*
Caliothrips phaseoli	*Oxyopes salticus*
Neohydatothrips gracilipes	*Oxyopes salticus*
Echinothrips mexicanus	*Oxyopes salticus*
Stomatothrips angustipennis	*Oxyopes salticus*
Franklinothrips vespiformis	*Oxyopes salticus*
Liriomyza trifolii	*Oxyopes salticus*
Empoasca kraemeri	*Oxyopes salticus*
Toxomerus procrastinatus	*Oxyopes salticus*
Condylostylus erectus	*Oxyopes salticus*
Aphidoletes sp	*Oxyopes salticus*
Eriopsis conexa	*Oxyopes salticus*
Cycloneda sanguinea	*Oxyopes salticus*
Harpasus eversmanni	*Oxyopes salticus*
Harmonia axyridis	*Oxyopes salticus*
Hippodamia convergens	*Oxyopes salticus*
Sminthurus rosai	*Oxyopes salticus*
Tullbergia minensis	*Oxyopes salticus*
Lepidocyrtus pallidus	*Oxyopes salticus*
Entomobrya ataquensis	*Oxyopes salticus*
Seira sp	*Oxyopes salticus*
Arlea lucifuga	*Oxyopes salticus*
Sphaeridia biniserata	*Oxyopes salticus*
Xyonizius californicus	*Oxyopes salticus*
Isotes bertonii	*Oxyopes salticus*
Sternocolaspis quatuordeimcosta	*Oxyopes salticus*
Hortensia similis	*Oxyopes salticus*
Myzus persicae	*Hasarius adansoni*
Aulacorthum solani	*Hasarius adansoni*
Uroleucon ambrosiae	*Hasarius adansoni*
Empoasca kraemeri	*Hasarius adansoni*
Frankliniella schultzei	*Hasarius adansoni*
Caliothrips phaseoli	*Hasarius adansoni*
Neohydatothrips gracilipes	*Hasarius adansoni*
Echinothrips mexicanus	*Hasarius adansoni*
Stomatothrips angustipennis	*Hasarius adansoni*
Franklinothrips vespiformis	*Hasarius adansoni*
Diabrotica speciosa	*Hasarius adansoni*
Xyonizius californicus	*Hasarius adansoni*
Isotes bertonii	*Hasarius adansoni*
Sternocolaspis quatuordeimcosta	*Hasarius adansoni*
Hortensia similis	*Hasarius adansoni*

**Resource**	**Consumer**
Lagria villosa	*Hasarius adansoni*
Myzus persicae	*Menemerus bivittatus*
Aulacorthum solani	*Menemerus bivittatus*
Uroleucon ambrosiae	*Menemerus bivittatus*
Empoasca kraemeri	*Menemerus bivittatus*
Frankliniella schultzei	*Menemerus bivittatus*
Caliothrips phaseoli	*Menemerus bivittatus*
Neohydatothrips gracilipes	*Menemerus bivittatus*
Echinothrips mexicanus	*Menemerus bivittatus*
Stomatothrips angustipennis	*Menemerus bivittatus*
Franklinothrips vespiformis	*Menemerus bivittatus*
Diabrotica speciosa	*Menemerus bivittatus*
Sminthurus rosai	*Menemerus bivittatus*
Tullbergia minensis	*Menemerus bivittatus*
Lepidocyrtus pallidus	*Menemerus bivittatus*
Entomobrya ataquensis	*Menemerus bivittatus*
Seira sp	*Menemerus bivittatus*
Arlea lucifuga	*Menemerus bivittatus*
Sphaeridia biniserata	*Menemerus bivittatus*
Xyonizius californicus	*Menemerus bivittatus*
Isotes bertonii	*Menemerus bivittatus*
Sternocolaspis quatuordeimcosta	*Menemerus bivittatus*
Hortensia similis	*Menemerus bivittatus*
Lagria villosa	*Menemerus bivittatus*
Orius insidiosus	*Cheiracanthium inclusum*
Empoasca kraemeri	*Cheiracanthium inclusum*
Xyonizius californicus	*Cheiracanthium inclusum*
Isotes bertonii	*Cheiracanthium inclusum*
Sternocolaspis quatuordeimcosta	*Cheiracanthium inclusum*
Hortensia similis	*Cheiracanthium inclusum*
Empoasca kraemeri	*Polybia paulista*
Toxomerus procrastinatus	*Polybia paulista*
Condylostylus erectus	*Polybia paulista*
Aphidoletes sp	*Polybia paulista*
Diabrotica speciosa	*Polybia paulista*
Hortensia similis	*Polybia paulista*
Detritus	*Sminthurus rosai*
Detritus	*Tullbergia minensis*
Detritus	*Lepidocyrtus pallidus*
Detritus	*Entomobrya ataquensis*
Detritus	*Seira sp*
Detritus	*Arlea lucifuga*
Detritus	*Sphaeridia biniserata*
