# Supplementary material for: Stability lies in flowers: Plant diversification mediating shifts in arthropod food webs
Source: PLoS One. 2018 Feb 16;13(2):e0193045. doi: 10.1371/journal.pone.0193045 (PMC5815608; doi:10.1371/journal.pone.0193045)
Supplement: S3 Table — (PDF) [file pone.0193045.s003.pdf]

Flowers	Trophic level	Number of individuals/m²
0.00	0	9.42
0.00	0	0.00
0.00	0	9.42
0.00	0	0.00
0.00	0	0.00
0.00	0	9.42
0.00	0	0.00
0.00	0	9.42
0.00	0	18.84
0.00	0	0.00
0.00	0	9.42
0.00	0	9.42
0.00	0	9.42
0.00	0	9.42
0.00	0	18.84
0.33	0	18.84
0.00	0	18.84
0.00	0	18.84
0.33	0	47.10
0.67	0	28.26
0.00	0	37.68
0.67	0	28.26
0.33	0	47.10
0.33	0	28.26
3.00	0	18.84
3.00	0	9.42
1.67	0	28.26
3.00	0	18.84
3.67	0	28.26
2.00	0	18.84
7.33	0	37.68
7.67	0	28.26
4.33	0	18.84
8.00	0	37.68
7.00	0	18.84
7.33	0	37.68
14.33	0	47.10
15.67	0	37.68
16.67	0	56.52
18.33	0	56.52
17.67	0	56.52
15.33	0	47.10
21.67	0	56.52
25.67	0	65.94

Flowers	Trophic level	Number of individuals/m²
26.67	0	75.36
28.33	0	65.94
26.67	0	56.52
23.33	0	65.94
37.33	0	28.26
38.33	0	28.26
40.00	0	37.68
40.67	0	47.10
40.67	0	47.10
39.33	0	18.84
43.33	0	56.52
43.00	0	56.52
42.00	0	65.94
45.33	0	56.52
44.00	0	47.10
42.33	0	47.10
42.67	0	56.52
40.33	0	75.36
40.33	0	56.52
42.00	0	56.52
42.33	0	75.36
40.00	0	65.94
39.00	0	56.52
37.00	0	65.94
37.33	0	75.36
39.00	0	65.94
39.00	0	75.36
37.33	0	56.52
0.00	1	9.40
0.00	1	9.40
0.00	1	9.40
0.00	1	9.40
0.00	1	9.40
0.00	1	9.40
0.00	1	9.40
0.00	1	9.40
0.00	1	9.40
0.00	1	9.40
0.00	1	9.40
0.00	1	9.40
0.00	1	9.40
0.00	1	9.40
0.00	1	9.40
0.33	1	9.40

Flowers	Trophic level	Number of individuals/m²
0.00	1	9.40
0.00	1	9.40
0.33	1	9.40
0.67	1	9.40
0.00	1	9.40
0.67	1	9.40
0.33	1	9.40
0.33	1	9.40
3.00	1	9.40
3.00	1	9.40
1.67	1	9.40
3.00	1	9.40
3.67	1	9.40
2.00	1	9.40
7.33	1	9.40
7.67	1	9.40
4.33	1	9.40
8.00	1	9.40
7.00	1	9.40
7.33	1	9.40
14.33	1	9.40
15.67	1	9.40
16.67	1	9.40
18.33	1	9.40
17.67	1	9.40
15.33	1	9.40
21.67	1	9.40
25.67	1	9.40
26.67	1	9.40
28.33	1	9.40
26.67	1	9.40
23.33	1	9.40
37.33	1	9.40
38.33	1	9.40
40.00	1	9.40
40.67	1	9.40
40.67	1	9.40
39.33	1	9.40
43.33	1	9.40
43.00	1	9.40
42.00	1	9.40
45.33	1	9.40
44.00	1	9.40
42.33	1	9.40

Flowers	Trophic level	Number of individuals/m²
42.67	1	9.40
40.33	1	9.40
40.33	1	9.40
42.00	1	9.40
42.33	1	9.40
40.00	1	9.40
39.00	1	9.40
37.00	1	9.40
37.33	1	9.40
39.00	1	9.40
39.00	1	9.40
37.33	1	9.40
0.00	2	188.40
0.00	2	37.68
0.00	2	28.26
0.00	2	28.26
0.00	2	28.26
0.00	2	47.10
0.00	2	113.04
0.00	2	122.46
0.00	2	141.30
0.00	2	150.72
0.00	2	122.46
0.00	2	141.30
0.00	2	235.50
0.00	2	226.08
0.00	2	244.92
0.33	2	244.92
0.00	2	244.92
0.00	2	263.76
0.33	2	282.60
0.67	2	244.92
0.00	2	329.70
0.67	2	235.50
0.33	2	301.44
0.33	2	339.12
3.00	2	28.26
3.00	2	28.26
1.67	2	56.52
3.00	2	56.52
3.67	2	47.10
2.00	2	37.68
7.33	2	65.94
7.67	2	75.36

Flowers	Trophic level	Number of individuals/m²
4.33	2	56.52
8.00	2	84.78
7.00	2	65.94
7.33	2	56.52
14.33	2	122.46
15.67	2	103.62
16.67	2	94.20
18.33	2	113.04
17.67	2	65.94
15.33	2	94.20
21.67	2	65.94
25.67	2	94.20
26.67	2	75.36
28.33	2	84.78
26.67	2	84.78
23.33	2	103.62
37.33	2	47.10
38.33	2	28.26
40.00	2	37.68
40.67	2	37.68
40.67	2	37.68
39.33	2	47.10
43.33	2	103.62
43.00	2	75.36
42.00	2	94.20
45.33	2	103.62
44.00	2	94.20
42.33	2	113.04
42.67	2	103.62
40.33	2	113.04
40.33	2	122.46
42.00	2	113.04
42.33	2	103.62
40.00	2	131.88
39.00	2	75.36
37.00	2	75.36
37.33	2	113.04
39.00	2	84.78
39.00	2	75.36
37.33	2	113.04
0.00	3	0.00
0.00	3	0.00
0.00	3	0.00
0.00	3	0.00

Flowers	Trophic level	Number of individuals/m²
0.00	3	0.00
0.00	3	0.00
0.00	3	0.00
0.00	3	0.00
0.00	3	0.00
0.00	3	0.00
0.00	3	0.00
0.00	3	0.00
0.00	3	0.00
0.00	3	0.00
0.00	3	0.00
0.33	3	0.00
0.00	3	0.00
0.00	3	0.00
0.33	3	0.00
0.67	3	9.42
0.00	3	9.42
0.67	3	0.00
0.33	3	0.00
0.33	3	0.00
3.00	3	0.00
3.00	3	28.26
1.67	3	9.42
3.00	3	18.84
3.67	3	0.00
2.00	3	9.42
7.33	3	9.42
7.67	3	0.00
4.33	3	9.42
8.00	3	37.68
7.00	3	9.42
7.33	3	18.84
14.33	3	28.26
15.67	3	47.10
16.67	3	37.68
18.33	3	47.10
17.67	3	56.52
15.33	3	37.68
21.67	3	37.68
25.67	3	56.52
26.67	3	75.36
28.33	3	28.26
26.67	3	28.26
23.33	3	94.20

Flowers	Trophic level	Number of individuals/m²
37.33	3	28.26
38.33	3	47.10
40.00	3	56.52
40.67	3	18.84
40.67	3	28.26
39.33	3	28.26
43.33	3	37.68
43.00	3	28.26
42.00	3	84.78
45.33	3	47.10
44.00	3	65.94
42.33	3	47.10
42.67	3	75.36
40.33	3	75.36
40.33	3	84.78
42.00	3	103.62
42.33	3	56.52
40.00	3	37.68
39.00	3	56.52
37.00	3	65.94
37.33	3	28.26
39.00	3	56.52
39.00	3	75.36
37.33	3	37.68
0.00	4	0.00
0.00	4	0.00
0.00	4	0.00
0.00	4	9.42
0.00	4	0.00
0.00	4	0.00
0.00	4	9.42
0.00	4	0.00
0.00	4	0.00
0.00	4	0.00
0.00	4	0.00
0.00	4	0.00
0.00	4	0.00
0.00	4	0.00
0.00	4	0.00
0.33	4	9.42
0.00	4	0.00
0.00	4	9.42
0.33	4	18.84
0.67	4	0.00

Flowers	Trophic level	Number of individuals/m²
0.00	4	9.42
0.67	4	18.84
0.33	4	9.42
0.33	4	9.42
3.00	4	0.00
3.00	4	9.42
1.67	4	0.00
3.00	4	0.00
3.67	4	0.00
2.00	4	0.00
7.33	4	18.84
7.67	4	9.42
4.33	4	9.42
8.00	4	0.00
7.00	4	18.84
7.33	4	0.00
14.33	4	9.42
15.67	4	9.42
16.67	4	0.00
18.33	4	9.42
17.67	4	0.00
15.33	4	18.84
21.67	4	9.42
25.67	4	9.42
26.67	4	0.00
28.33	4	28.26
26.67	4	37.68
23.33	4	9.42
37.33	4	9.42
38.33	4	0.00
40.00	4	0.00
40.67	4	0.00
40.67	4	18.84
39.33	4	18.84
43.33	4	0.00
43.00	4	28.26
42.00	4	9.42
45.33	4	9.42
44.00	4	18.84
42.33	4	18.84
42.67	4	18.84
40.33	4	0.00
40.33	4	28.26
42.00	4	0.00

Flowers	Trophic level	Number of individuals/m²
42.33	4	28.26
40.00	4	56.52
39.00	4	18.84
37.00	4	0.00
37.33	4	37.68
39.00	4	9.42
39.00	4	9.42
37.33	4	47.10
0.00	5	0.00
0.00	5	0.00
0.00	5	0.00
0.00	5	0.00
0.00	5	0.00
0.00	5	0.00
0.00	5	0.00
0.00	5	0.00
0.00	5	0.00
0.00	5	9.42
0.00	5	0.00
0.00	5	0.00
0.00	5	0.00
0.00	5	18.84
0.00	5	9.42
0.33	5	0.00
0.00	5	0.00
0.00	5	9.42
0.33	5	0.00
0.67	5	0.00
0.00	5	0.00
0.67	5	0.00
0.33	5	9.42
0.33	5	9.42
3.00	5	0.00
3.00	5	0.00
1.67	5	9.42
3.00	5	9.42
3.67	5	0.00
2.00	5	0.00
7.33	5	0.00
7.67	5	9.42
4.33	5	0.00
8.00	5	9.42
7.00	5	0.00
7.33	5	0.00

Flowers	Trophic level	Number of individuals/m²
14.33	5	0.00
15.67	5	0.00
16.67	5	9.42
18.33	5	0.00
17.67	5	0.00
15.33	5	0.00
21.67	5	9.42
25.67	5	18.84
26.67	5	0.00
28.33	5	0.00
26.67	5	0.00
23.33	5	0.00
37.33	5	9.42
38.33	5	0.00
40.00	5	0.00
40.67	5	9.42
40.67	5	0.00
39.33	5	0.00
43.33	5	9.42
43.00	5	9.42
42.00	5	0.00
45.33	5	0.00
44.00	5	0.00
42.33	5	9.42
42.67	5	0.00
40.33	5	0.00
40.33	5	9.42
42.00	5	9.42
42.33	5	0.00
40.00	5	0.00
39.00	5	0.00
37.00	5	0.00
37.33	5	0.00
39.00	5	9.42
39.00	5	0.00
37.33	5	0.00
